# ESRP2 controls an adult splicing programme in hepatocytes to support postnatal liver maturation

**DOI:** 10.1038/ncomms9768

**Published:** 2015-11-04

**Authors:** Amruta Bhate, Darren J. Parker, Thomas W. Bebee, Jaegyoon Ahn, Waqar Arif, Edrees H. Rashan, Sandip Chorghade, Anthony Chau, Jae-Hyung Lee, Sayeepriyadarshini Anakk, Russ P. Carstens, Xinshu Xiao, Auinash Kalsotra

**Affiliations:** 1 Department of Biochemistry and Medical Biochemistry, University of Illinois, Champaign, Illinois 61801, USA; 2 Departments of Medicine and Genetics, Perelman School of Medicine, University of Pennsylvania, Philadelphia, Pennsylvania 19104, USA; 3 Department of Integrative Biology and Physiology, University of California, Los Angeles, California 90095-7246, USA; 4 Department of Molecular and Integrative Physiology, University of Illinois, Champaign, Illinois 61801, USA; 5 Carl R. Woese Institute of Genomic Biology, University of Illinois, Champaign, Illinois 61801, USA

## Abstract

Although major genetic networks controlling early liver specification and morphogenesis are known, the mechanisms responsible for postnatal hepatic maturation are poorly understood. Here we employ global analyses of the mouse liver transcriptome to demonstrate that postnatal remodelling of the liver is accompanied by large-scale transcriptional and post-transcriptional transitions that are cell-type-specific and temporally coordinated. Combining detailed expression analyses with gain- and loss-of-function studies, we identify epithelial splicing regulatory protein 2 (ESRP2) as a conserved regulatory factor that controls the neonatal-to-adult switch of ∼20% of splice isoforms in mouse and human hepatocytes. The normal shift in splicing coincides tightly with dramatic postnatal induction of ESRP2 in hepatocytes. We further demonstrate that forced expression of ESRP2 in immature mouse and human hepatocytes is sufficient to drive a reciprocal shift in splicing and causes various physiological abnormalities. These findings define a direct role for ESRP2 in the generation of conserved repertoires of adult splice isoforms that facilitate terminal differentiation and maturation of hepatocytes.

## Abstract (web summary)

Alternative RNA splicing is important during organismal development. Here, the authors perform RNA-Seq on mouse and human liver samples to provide a comprehensive view of splicing events during liver development and growth, and identify Espr2 as a main regulator of these splicing processes.

## Graphical abstract

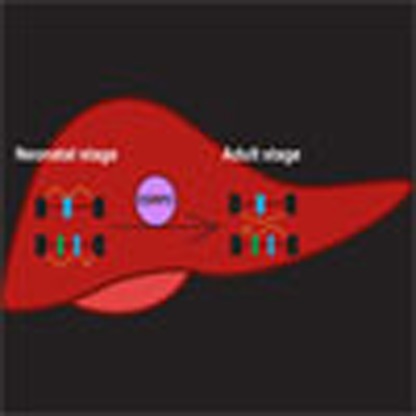

Mammalian tissues initially form and begin functioning in the embryo but are extensively remodelled after birth to rapidly adapt and perform adult functions. This process is especially true for the liver, which is haematopoietic in the embryo but converts into a major metabolic tissue in the adult^1^. Hepatocytes, which are highly proliferative in the fetus, become quiescent, undergo hypertrophic growth and mature via large-scale changes in gene expression to maintain metabolic homoeostasis during the dramatic transitions that occur during and after birth. Diverse genetic mechanisms ensure that these changes occur precisely and coordinately to initiate proper lineage specification, cell growth and differentiation^2^. Most gene regulation studies in the liver have focused on transcriptional control^3^,^4^; however, it is becoming clear that post-transcriptional mechanisms such as alternative pre-mRNA splicing (AS) have essential roles in sequential replacement of fetal-to-adult protein isoforms^5^,^6^,^7^,^8^,^9^.

AS allows multiple mRNAs with potentially different functions to be produced from a single gene^10^,^11^. Several estimates indicate that >95% of human multi-exon genes are alternatively spliced^12^,^13^, and that most are extensively regulated in response to physiological needs^14^. Such exquisite control is exerted through multiple RNA-binding proteins that bind to ‘core’ and ‘auxiliary’ elements on pre-mRNAs to influence assembly of the basal splicing machinery near the 5′ and 3′ splice sites^15^,^16^,^17^,^18^,^19^. The key splicing regulators that orchestrate tissue-specific AS programmes in brain^20^,^21^,^22^,^23^,^24^,^25^, heart^26^,^27^,^28^,^29^,^30^,^31^,^32^, skeletal muscle development^33^,^34^,^35^,^36^,^37^ or T-cell activation^38^,^39^,^40^ are well characterized; however, neither the full extent of transcript diversity nor the regulatory factors that drive isoform transitions in liver development are known.

Here we take a systematic approach to identify a highly conserved and temporally coordinated cell-type-specific splicing programme, which is activated in part by epithelial splicing regulatory protein 2 (ESRP2) during postnatal period of liver development. Consistent with the failure of many neonatal-to-adult splicing transitions, *Esrp2* null mice exhibit persistent expression of fetal markers and diminished mature hepatocyte characteristics. Conversely, ectopic expression of ESRP2 in immature mouse and human hepatocytes results in a reciprocal switch in splicing of genes involved in cell proliferation, adhesion and differentiation. Phenotypic characterization of *Esrp2* null livers reveals defects in hepatocyte proliferation, hepatic zonation abnormalities and reduction in albumin production. Thus, our results define a conserved ESRP2 splicing regulatory network that supports terminal differentiation and postnatal maturation of hepatocytes.

## Results

### Extensive transcriptome remodelling during liver maturation

**Figure 1:**
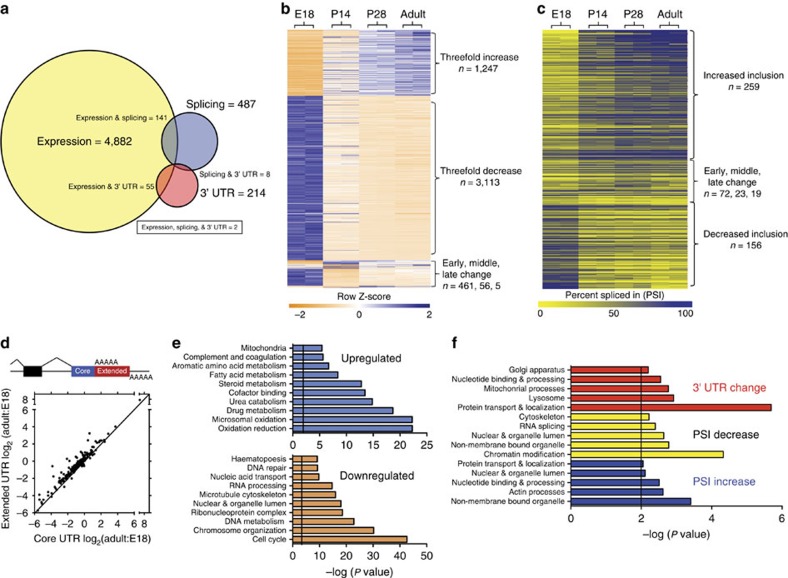
Remodelling of the liver transcriptome during postnatal development. (**a**) Venn diagram showing overlap of genes that change in mRNA abundance (>3-fold; *P* value<0.01, Fisher’s exact test), alternative splicing (PSI>20%), and/or 3′UTR length ratio (>20%). (**b**) Heat map representation of 4,882 genes that change significantly in expression from E18, P14, P28 and adult mouse liver developmental time points. Genes with early, middle and late changes showed a significant difference in expression only at one time point. (**c**) Heat map representation of 529 splicing changes across development. (**d**) Scatterplot of core and extended 3′UTR changes in adult and E18 mouse livers (>20% change). Gene ontology analysis for significantly enriched pathways for genes (**e**) increasing and decreasing in mRNA abundance, (**f**) increasing or decreasing in PSI, and/or changing their 3′UTR length. *P* value=0.01 is shown with a black line, Fisher’s exact *t*-test.

To identify global changes in the liver transcriptome during postnatal development, we performed a high-resolution RNA-seq analysis on poly (A)-selected RNA in biological duplicates from four developmental time points in the mouse liver: embryonic day (E)18, postnatal day (P)14, P28, and adult. We obtained an average of 200 million paired-end 100 base pair (bp) reads, with at least 88% mapped to the mouse genome (Supplementary Table 1). The majority of the transcriptome changes were in mRNA abundance, as we identified 4,882 differentially expressed genes between E18 and adult (>3.0 fold, Fig. 1a). Comparative analysis of mRNA isoforms identified 529 AS events across 487 unique genes whose percent spliced in (PSI) values changed >20% (ΔPSI>20%), whereas 214 genes exhibited a >20% change in alternative polyadenylation (APA). A pie chart distribution of different types of AS is shown in the Supplementary Fig. 1a. We tested 179 developmentally regulated AS events from RNA-seq using reverse transcription PCR (RT–PCR) and validated 151 (∼84%) of them (Supplementary Fig. 1b–d; Supplementary Data 1). Most AS events (58%) were multiples of three nucleotides, indicating variably spliced regions in the liver tend to preserve the reading frame.

Remarkably, the overlap between mRNA abundance, AS and APA was marginal as only 141 genes (29% of AS events) changed at both steady-state mRNA levels and splicing, 55 genes (26% of 3′ untranslated region (UTR) changes) changed at both mRNA levels and 3′UTR length, and eight genes changed in AS and in 3′UTR length (Fig. 1a). Of the 5,583 total events, only two changed significantly in mRNA levels, AS and 3′UTR length. Notably, gene ontology analyses of the transcripts undergoing changes in mRNA abundance, AS or 3′UTR length revealed enrichment in unique functional categories. For instance, the up- or downregulated genes showed strong enrichment for ‘metabolic’ or ‘cell cycle’ related gene ontology functions whereas the alternatively spliced genes with increased or decreased PSI values showed enrichment for ‘actin processes’ or ‘chromatin modification’, respectively (Fig. 1e,f). The class of genes with changes in APA showed particular over-representation for ‘protein transport and localization’ functions amongst enrichment for lysosomal and mitochondrial processes (Fig. 1f). RNA-seq analysis also showed that nearly three times as many genes decreased in abundance than increased during development (Fig. 1b). While some genes changed significantly at either an early, middle or late stage, most genes fell into a distinct category of either continual increase or decrease from E18 to adult. AS transitions also showed similar temporal clusters. We found that nearly twice as many variably spliced regions showed increased inclusion than decreased over the postnatal period (Fig. 1c). The strongest directional pattern of change, however, was in the 3′UTR length through APA. Of the 214 affected genes, 145 (68%) showed preferential usage of distal poly (A) sites, thereby generating isoforms with longer 3′UTRs (Fig. 1d).

### Conservation of AS transitions in murine and human livers

**Figure 2:**
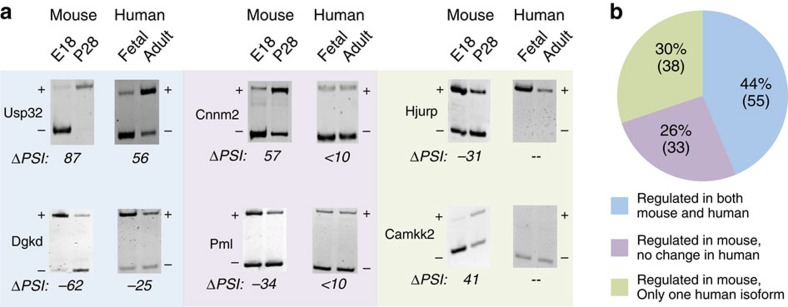
Conservation of developmentally regulated AS in mouse and human livers. (**a**) Examples of mouse and human AS during liver development. The band corresponding to (+) indicates exon inclusion and (−) indicates exon skipping. Gene names are indicated on the left, and the differences in percent spliced in (ΔPSI) values are shown below each image. (**b**) Pie chart distribution showing splicing conservation in mouse and human liver development. A total of 126 splicing events were tested side-by-side with ΔPSI values over 20% in mouse and 10% in human deemed significant.

To determine if the developmental AS transitions identified in mouse liver were conserved in humans, we directly assessed splicing of 126 homologous regions in fetal and adult human liver samples (Supplementary Data 2). The bulk (55, 44%) of the AS events tested were found to be similarly regulated in both species (Fig. 2a,b). Another 33 (26%) events were regulated in mouse, but did not show a developmental change in humans, while 38 (30%) events that were alternatively spliced and regulated in mouse were either constitutively included or skipped in humans. The regulated exons in both species showed higher nucleotide level conservation on average than exons that were constitutive in humans (Supplementary Fig. 2a). As was witnessed in the mouse liver, the majority (78%) of AS changes in humans showed increased inclusion across time (Supplementary Fig. 2b). Collectively, these results not only demonstrate a strong correlative directionality in developmental regulation of AS events but also prelude a functional significance for regulated splicing transitions in hepatic maturation.

### Postnatal shift in AS is cell-type-specific and temporally coordinated

The liver is comprised of many different cells, but hepatocytes are the major parenchymal cell type and account for >75% of the adult liver volume^1^. Hepatocytes are epithelial in nature and form branching plates of cells between capillary sinusoids that connect the portal tracts to the central vein. The non-parenchymal cells (NPC) include cholangiocytes, hepatic stellate cells, sinusoidal endothelial cells and Kupffer cells. Notably, the fetal liver functions as a hematopoietic organ in mammals; however, by the end of gestation the haematopoietic progenitors exit the liver and migrate to the bone marrow^1^. Therefore, we asked whether the transcriptome changes identified were exclusive to the maturing parenchyma of the liver or were arising due to varying cell populations.

**Figure 3:**
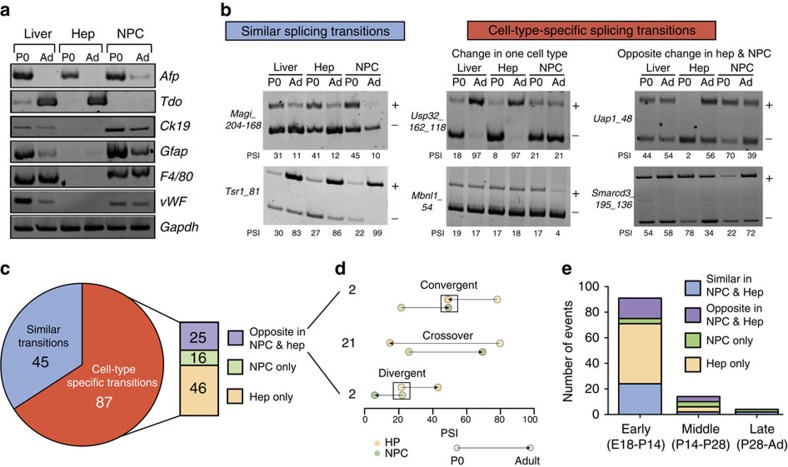
Cell-type-specificity of AS programme during postnatal liver maturation. (**a**) RT–PCR assays of cell-type-specific markers *Afp* (neonatal stage), *Tdo* (adult Hep), *Ck19* (cholangiocytes), *Gfap* (stellate cells), *F4/80* (Kupffer cells), *vWF* (endothelial cells) and *Gapdh* (all cells) on RNA isolated from postnatal day 0 (P0) and adult (Ad) whole liver, hepatocytes (Hep) and non-parenchymal cells (NPC). (**b**) Representative gel images for the developmentally regulated splicing events in whole liver, Hep and NPC fractions. (**c**) Pie chart summarizing the breakdown of ‘similar’ versus ‘cell type specific’ splicing transitions. Bar graph showing the number of events regulated only in Hep, NPC or in opposite direction. Individual splicing events were repeated on at least three independent pools of samples. (**d**) Classification and directionality of oppositely regulated splicing transitions during hepatocyte and NPC maturation. (**e**) Temporal dynamics of splicing transitions in hepatocytes and NPCs. Bar graph shows the proportion of cell-type-specific transitions that follow the early, middle or late patterns of splicing change during liver maturation.

To distinguish between the two scenarios, we isolated hepatocytes and NPC fractions from livers of P0 and adult Friend Virub B NIH Jackson (FVB/NJ) mice by enzymatic digestion and differential centrifugation. Total RNAs were extracted from the two liver cell fractions within 1 h of animal sacrifice to minimize the effects of post-mortem changes and cell manipulation. Relative purity of the fractions was determined by RT–PCR analysis of cell-type-specific markers (Fig. 3a). Comparisons of 132 AS events from purified hepatocytes, NPC and whole liver across two developmental stages revealed that the vast majority (∼88%) of changes are detected in hepatocytes (Fig. 3b,c; Supplementary Data 3). We extended our AS analysis to further determine the cell-type-specificity. We found that while a substantial number of events (45, 34%) undergo similar postnatal transitions in hepatocyte and NPC fractions, a much larger set of events (87, 66%) undergo transitions that are cell type specific (Fig. 3b,c; Supplementary Data 3). We further classified the AS events on the basis of whether the postnatal splicing change occurred in hepatocyte only, NPC only or opposite in NPC and hepatocyte fractions. Individual examples representing each of these categories are shown in Fig. 3b. Although more than half of the cell-type-specific transitions (53%) were exclusive to hepatocytes, a significant number of events changed specifically in NPC (18%) or reciprocally in NPC and hepatocyte fractions (29%). This implies that postnatal transitions in the liver are primarily represented by regulation within hepatocytes and are not a consequence of change in cell populations.

While analysing the directionality of the opposite in NPC and hepatocyte AS transitions, we noticed that 21 of 25 events displayed a crossover pattern (Fig. 3d; Supplementary Data 3). That is, if the hepatocyte fraction showed a decrease in PSI of a variable region between P0 and adult, the NPC fraction showed an increase and vice versa. In contrast, 2 of 25 events exhibited a convergent pattern. That is, while the PSI value of the variable regions at P0 stage was different between the hepatocyte and NPC fractions; their adult PSI values became indistinguishable (Fig. 3d). Two of 25 events showed a strong divergent pattern. That is, the PSI value of the variable region at P0 stage was comparable in hepatocyte and NPC fractions but their adult PSI value diverged in reciprocal directions (Fig. 3d). Furthermore, we determined the temporal dynamics of cell-type-specific splicing differences (Fig. 3e; Supplementary Data 3). We found that most AS transitions, especially the hepatocyte-specific events, happen early (maximal change occurs between E18 and P14) and that few events follow the middle (maximal change between P14 and P28) and late patterns (maximal change between P28 and adult) of splicing change. Together, these results highlight the remarkable complexity of the transcriptome that arises in functionally distinct nonetheless highly interacting liver cell populations through postnatal development.

### ESRP2 is upregulated in hepatocytes during liver maturation

**Figure 4:**
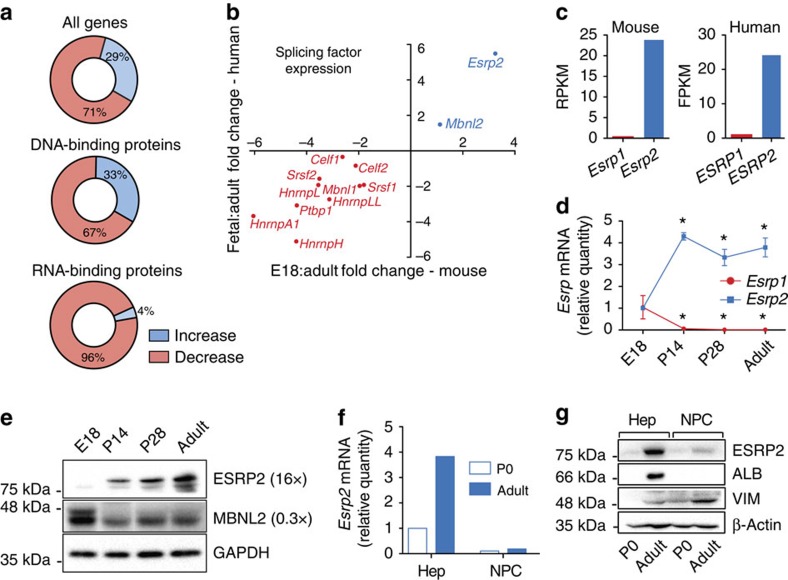
Hepatocyte-specific postnatal upregulation of *Esrp2* mRNA and protein levels. (**a**) Doughnut chart distribution showing number of genes increasing or decreasing in mRNA abundance (>3-fold) during mouse liver development (E18–Adult). The lists of nucleic acid binding proteins were obtained from the AmiGO 2 database. Direct comparison of ‘All genes’ (4,882) to sequence specific ‘DNA binding’ (83) and ‘RNA binding’ (313) proteins show a larger proportion of RNA-binding proteins is downregulated. (**b**) Comparative analyses of transcript levels (qRT–PCR) of select auxiliary splicing factors during mouse (*x* axis) and human (*y* axis) liver development. Each data point represents fold change in steady-state mRNA levels of individual splicing factor relative to *Gapdh*. (**c**) Relative *Esrp1* and *Esrp2* mRNA levels in mouse and human adult livers. FPKM values for human ESRP1 and ESRP2 were obtained from the Human Protein Atlas Database (www.proteinatlas.org). (**d**) qRT–PCR analysis of *Esrp1* and *Esrp2* mRNA expression during mouse postnatal liver maturation. Each data point was normalized to *Gapdh* and represents fold change (mean±s.d.) relative to E18. *P<0.05, Student’s *t*-test (*n*=3). (**e**) Western blot showing induced ESRP2 and reduced MBNL2 steady-state protein levels during mouse liver development. Fold changes calculated by quantification of relative band intensities normalized to GAPDH are shown in parentheses on the right. Relative quantity and cell-type-specific postnatal increase of (**f**) *Esrp2* mRNA and (**g**) ESRP2 protein in Hep and NPC fractions.

Our next goal was to identify the regulatory factor(s) that drive postnatal AS transitions in the liver. Whole liver RNA-seq analyses revealed strong enrichment of ribonucleoprotein complex and RNA processing functions in the downregulated gene set (Fig. 1e). Genes were cross-referenced to the RNA-seq data to identify DNA- and RNA-binding proteins that change >3-fold in expression during postnatal liver maturation. Direct comparison revealed that an overwhelming majority of RNA-binding proteins (96%) decreased postnatally (Fig. 4a). In contrast, sequence-specific DNA binding proteins showed a similar overall pattern as ‘All genes’. qRT–PCR analysis of select RNA-binding proteins known to directly regulate splicing^18^ demonstrated a high degree of overlap in their developmental expression pattern between mouse and human (Fig. 4b). Consistent with the RNA-seq data, qRT–PCR results confirmed a strong decrease in hepatic mRNA levels for most auxiliary splicing factors tested during development (Fig. 4b).

*Esrp2* and Muscleblind-like 2 (*Mbnl2*) exhibited significant mRNA upregulation in mouse and human liver development (Fig. 4b). ESRPs (ESRP1 and ESRP2) are splicing regulators that display an overlapping expression pattern in epithelial cells. They drive mesenchymal-to-epithelial transition by coordinating splicing of genes involved in cell–cell adhesion, cytoskeleton rearrangement and intracellular signalling^41^,^42^. We found that in comparison to *Esrp1*, *Esrp2* is the primary paralogue expressed in both mouse and human livers (Fig. 4c; Supplementary Fig. 3a). This is different from most other epithelial tissues where ESRP1 is more abundant^43^. Furthermore, the developmental time course revealed a reciprocal expression pattern: *Esrp2* mRNA levels increased while *Esrp1* levels declined postnatally (Fig. 4d).

Parallel analysis of protein levels revealed a striking increase in ESRP2 during the first 4 weeks after birth (Fig. 4e). ESRP2 protein levels in the adult mouse liver are ∼16-fold higher in comparison to the E18 stage. Despite a modest increase in transcript levels (Fig. 4b), MBNL2 protein levels actually declined by approximately 3-fold in the adult liver (Fig. 4e). As with mRNA expression, ESRP1 protein is undetectable in the adult human and mouse livers (Supplementary Fig. 3b). To determine whether the increase in *Esrp2* expression is cell-type-specific, we assayed its relative mRNA and protein levels in purified hepatocyte and NPC fractions isolated from pooled P0 and adult mouse livers. We found that *Esrp2* transcript levels increased 4-fold in adult hepatocytes versus the P0 stage (Fig. 4f). Western blot analysis demonstrated a corresponding increase in ESRP2 protein levels, specifically in adult hepatocytes (Fig. 4g). These results illustrate that *Esrp2* expression is specifically induced in the maturing hepatocytes, and that the timing of induction coincides with the developmental period (E18–P14) when most postnatal AS transitions occur (Figs 3e and 4d,e).

### ESRP2 controls postnatal hepatic AS transitions

**Figure 5:**
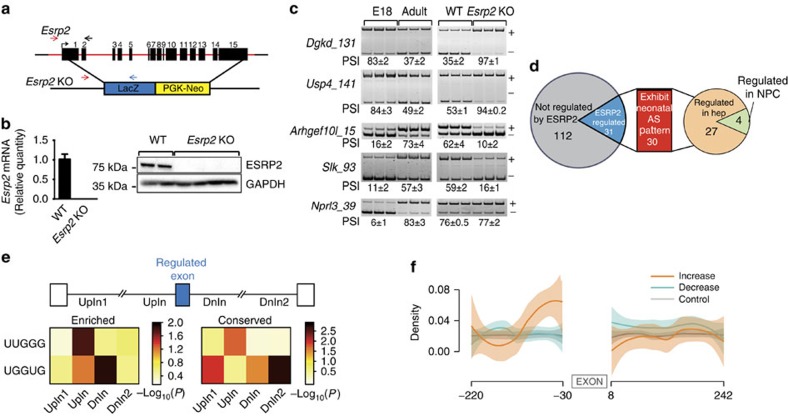
*Esrp2* ablation leads to failure of postnatal AS transitions in the liver. (**a**) Schematic showing the full *Esrp2* gene replaced by homologous recombination to generate a knockout mouse. Coloured arrows indicate the positions of primers designed to determine the genotype of Wild-type and *Esrp2* KO mice. (**b**) qRT–PCR analysis showing complete loss of *Esrp2* mRNA in liver tissue from *Esrp2* knockout (KO) mice. Data (mean±s.d., *n*=4) were normalized to *Gapdh* (left). Western blot analysis demonstrating complete loss of ESRP2 in *Esrp2* knockout livers. GAPDH was used as loading control (right). (**c**) Representative gel images for the developmentally regulated splicing events (E18 versus adult; FVB/NJ strain) in the adult wild-type and *Esrp2* KO livers (C57BL/6 strain). Four representative splicing events that exhibit failure of neonatal-to-adult switch, and one representative event that remains unchanged in *Esrp2* KO livers. (**d**) Pie–bar–pie representation showing number of ESRP2-regulated splicing events that exhibit a developmental shift towards the neonatal pattern in *Esrp2* KO livers, and their cell type specificity during normal development. (**e**) Conservation (eight mammalian species including human) and enrichment of ESRP-binding motifs in the flanking introns of the ESRP2-regulated splicing events. Heat maps representing *P* values for significance of indicated motifs in positions 12 to 250 of the upstream intron (upIn1), positions −250 to −31 of the upstream intron (upIn), positions 12 to 250 of the downstream intron (dnIn), and positions −250 to −31 of the downstream intron (dnIn2) are shown on the right. *P* values were calculated using binomial distribution (**f**) Enriched ESRP2 motifs upstream and downstream (±150 bp) of ESRP2-sensitive alternative regions were identified. The light blue, orange and grey lines indicate the distribution of the UGG core motif around the ESRP2-regulated alternative regions, which decrease, increase or show no change in *Esrp2* KO livers, respectively.

To investigate whether ESRP2 plays a determinative role in the neonatal-to-adult shift of AS in the liver, we generated *Esrp2* knockout (KO) mice (Fig. 5a). Complete loss of *Esrp2* mRNA and protein expression in the livers of homozygous ESRP2 KOs is shown in Fig. 5b. We next determined the AS pattern of 143 developmentally regulated events in adult livers of *Esrp2* KOs and wild-type littermate controls. Thirty-one events were strongly misspliced in *Esrp2* KOs of which 68% exhibited increased skipping and 32% increased inclusion (Supplementary Data 4). Representative examples demonstrating complete dependence of select postnatal splicing transitions in *Esrp2* KO livers are shown in Fig. 5c. In fact, over 96% of ESRP2-regulated events retained the neonatal splicing pattern in adult KO livers, indicating ESRP2 is obligatory for these physiological switches (Fig. 5d).

When evaluated for cell-type-specificity, 87% of ESRP2 targets were primarily regulated in hepatocytes (Fig. 5d; Supplementary Data 3). We found that these targets are significantly enriched for gene ontology terms related to actin cytoskeleton and Rho/Ras signalling pathways (Supplementary Fig. 4a). Protein–protein interaction analysis revealed a connected network among targets with functional enrichment for ‘epithelial cell differentiation’ and ‘phosphate containing metabolic process’ (Supplementary Fig. 4b). ESRP-binding motif UUGGG was significantly enriched in the upstream introns whereas the UGGUG motif was both enriched and conserved in the downstream introns flanking the ESRP2-regulated exons (Fig. 5e). Furthermore, we observed prominent enrichment of the core ESRP motif immediately upstream of the variable regions whose PSI values increased in *Esrp2* KO livers, compared with those with unchanged PSI values (Fig. 5f). These results agree with the previous observation that ESRP-binding upstream of the regulated exon represses its inclusion^41^.

**Figure 6:**
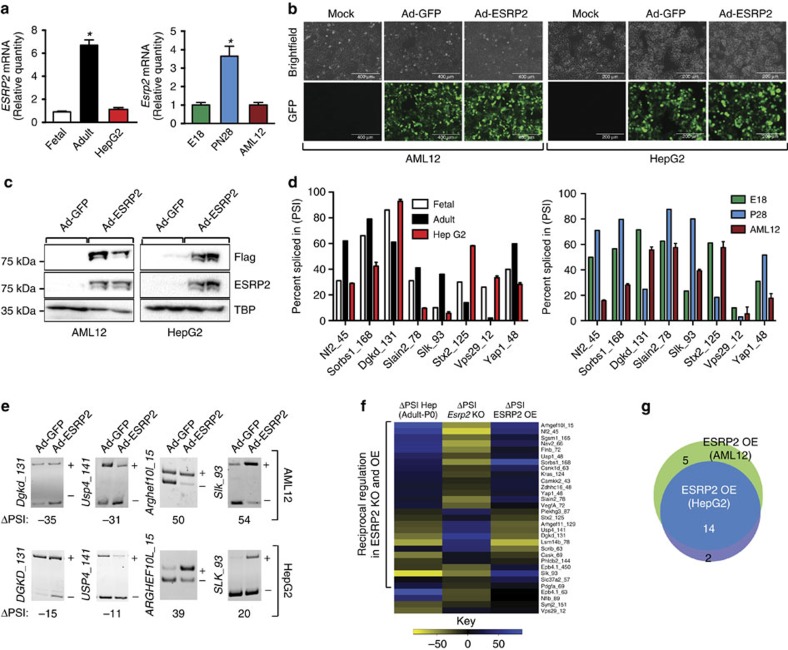
ESRP2 regulates a conserved set of postnatal AS transitions in hepatocytes. (**a**) qRT–PCR analysis of *ESRP2* mRNA levels in fetal, adult human liver samples, and HepG2 cells (left panel); E18, adult mouse liver samples and AML12 cells (right panel). mean±s.d. (**b**) Adenoviral overexpression of GFP and ESRP2 in AML12 and HepG2. (**c**) Western blot demonstrating increased expression of FLAG-tagged ESRP2 in AML12 and HepG2 cells infected with the adenovirus. Scale bars, 400 μm (**d**) Splicing assays of ESRP2 target genes using human fetal, adult liver tissue samples, and HepG2 cells (left panel); E18, adult mouse liver tissue, and AML12 cells (right panel). (**e**) Four representative AS events that exhibit neonatal-to-adult shift in splicing upon adenoviral ESRP2 overexpression in AML12 and HepG2 cells. (**f**) Heat map of ΔPSI values (Adult–P0) of AS events in Hepatocytes/NPCs with an overlap between AS events that exhibit reciprocal regulation between *Esrp2* KO livers and ESRP2 overexpression in AML12 cells. (**g**) Venn diagram of conserved AS regulation between AML12 and HepG2 cells upon ESRP2 overexpression.

Next, we determined whether ESRP2-regulated AS events are conserved between mouse and human hepatocytes. For this, we used human HepG2 and mouse AML12 hepatic cell lines, which have very low basal *ESRP2* mRNA and protein levels and express a neonatal AS programme (Fig. 6a–c). Strikingly, forced expression of ESRP2 reciprocally shifted AS from neonatal-to-adult patterns in both AML12 (24 of 31, 77%) and HepG2 (16 of 21, 76%) cells (Fig. 6d–f; Supplementary Data 4). A highly significant (*P*<2.664e^−10^, two-sample test for equality of proportions) negative correlation (*R*=−0.87, Pearson’s product–momentum correlation) between *Esrp2* KO and overexpression results indicates that developmentally regulated splicing of a subset of pre-mRNAs is extremely sensitive to ESRP2 levels (Fig. 6f). Furthermore, ESRP2-regulated AS events exhibit significantly higher mouse-to-human conservation (67%, *P*<0.05, two-sample test for equality of proportions) in terms of pattern and directionality (Fig. 6g) when compared with background (44%, Fig. 1b). Overall, these results provide compelling evidence that ESRP2 is a conserved regulatory factor that is both necessary and sufficient to stimulate a subset of postnatal splicing transitions in hepatocytes.

### ESRP2 ablation causes defects in hepatic maturation and function

**Figure 7:**
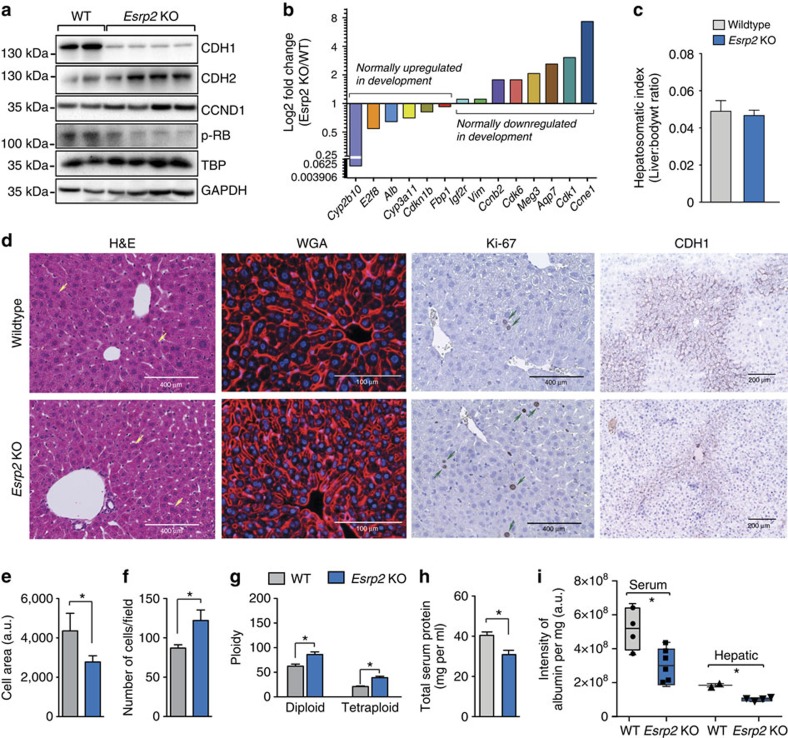
Histological and functional defects in the livers of *Esrp2* KO mice. (**a**) Western blot analysis demonstrating a decrease in E-cadherin (CDH1) and Retinoblastoma (pRB) and an increase in N-cadherin (CDH2) and Cyclin D1 (CCND1) protein expression in *Esrp2* KO livers. GAPDH and TBP were used as loading controls. (**b**) qRT–PCR analysis of cell cycle regulators as well as neonatal and adult hepatocyte markers in wild type (WT) and *Esrp2* KO livers (*n*=3). (**c**) Bar graph of hepatosomatic index of WT and *Esrp2* KO mice. mean±s.d. (**d**) Haematoxylin and eosin (H&E) staining of WT and *Esrp2* KO liver tissues. Yellow arrows indicate binucleated cells, scale bars, 400 μm; WGA- and DAPI-stained liver tissue sections representing cell size and number in WT and *Esrp2* KO mice, scale bars, 100 μm; increased number of Ki-67-positive cells in *Esrp2* KO livers (green arrows), scale bars, 400 μm; E-cadherin (CDH1) immunostaining measuring periportal zonation of WT and *Esrp2* KO livers, scale bars, 200 μm. Quantification of (**e**) hepatocyte cell area, mean±s.d.; (**f**) cell number, mean±s.d.; (**g**) ploidy, mean±s.d. (**h**) Bar graph showing a decrease in total serum protein levels in *Esrp2* KO (*n*=11) as compared with WT mice (*n*=6); mean±s.d. (**i**) Box-whisker plot demonstrating a decrease in serum and hepatic albumin levels in *Esrp2* KO (*n*=6) compared with WT mice (*n*=4), mean±s.d., *P*<0.005, Student’s *t*-test.

Consistent with the splicing defects, *Esrp2* KOs showed persistent expression of fetal genes and loss of mature hepatocyte markers including the EMT markers CDH1 and CDH2 (Fig. 7a,b). Particularly, we found that the transcript and protein levels of positive cell cycle regulators (*Ccnb2*, *Ccne1, Cdk1* and *Cdk6*; CCND1) are elevated and the levels of cell cycle inhibitors (*Cdkn1b and E2f8*; pRB) are reduced in the *Esrp2* KO livers (Fig. 7a,b). While the *Esrp2* KOs showed normal hepatosomatic index and lobular organization, these mice displayed a substantial increase in the number of diploid and tetraploid hepatocytes that were considerably smaller in size relative to their littermate controls (Fig. 7c–f). We also observed significantly higher hepatocyte proliferation in *Esrp2* KOs as evidenced by increased Ki-67 and phosphohistone 3 (pHIST3H3) staining (Fig. 7d,g; Supplementary Fig. 5a). *Esrp2* KOs did not exhibit any major signs of liver injury or changes in apoptosis, alanine aminotransferase, aspartate aminotransferase, cholesterol, triglyceride, or random and fasting glucose levels (Supplementary Fig. 5b,c). As some proteins during postnatal period develop region-specific expression along the portocentral axis^44^, we performed immunohistochemical localization analyses to examine the impact of *Esrp2* ablation on liver zonation. We found a significant reduction in periportal CDH1 and a relative expansion in the zone of PCK1 expressing periportal hepatocytes in the KO livers (Fig. 7d and Supplementary Fig. 6). While the restricted perivenous distribution of GLUL was maintained, the zone of CYP2E1 distribution was increased within the hepatic lobule of the KO livers (Supplementary Fig. 6). We also observed a significant decrease in total serum protein as well as serum and hepatic albumin levels in *Esrp2* KOs (Fig. 7h,i), which are likely due to decreased *Alb* mRNA abundance in the KOs. Together, these data demonstrate that ESRP2 serves an important role in terminal differentiation, hepatic zonation, and functional competence of hepatocytes.

## Discussion

Collectively, our results reflect that postnatal liver maturation is highly organized and is accomplished through at least three separate modes of transcriptome-remodelling events, namely mRNA abundance, AS and APA. We find different regulatory events target genes that function in more distinct than overlapping pathways. This signifies the discrete requirement of transcriptional and post-transcriptional regulatory modules in facilitating hepatic growth and development. Previous studies proposed that programmed changes in 3′UTR length by APA may affect mRNA stability of transcripts by changing the landscape of RNA-binding proteins and microRNAs available in a cell^45^. We demonstrate that mRNAs with altered 3′UTR isoform ratios are largely unaffected at steady-state levels and therefore argue against a major role for APA in determining mRNA abundance during liver maturation. These results are consistent with a recent study that found genes changing in mRNA expression during biological transitions tend to have single 3′UTRs^46^. In contrast, the multi-UTR genes during such transitions predominantly change their 3′UTR ratios to achieve tissue specificity^46^.

We also identified a conserved set of splice isoform transitions that switch from neonatal-to-adult pattern within the first two weeks after birth. While most of these AS transitions reflect changes in maturing hepatocytes, we discovered many are strictly NPC-specific. Of particular interest are the developmental transitions that when sampled in whole livers show no change in isoforms; however, when interrogated in purified hepatocytes versus NPC exhibit dramatic changes in isoforms but in opposite directions, which at the whole tissue level are diluted out. Intriguingly, majority of the hepatocyte-specific splicing switches happen during the period when the liver encounters extensive physiological changes. For example, in rodents, the first few weeks of postnatal development are associated with terminal differentiation of hepatocytes and pronounced increase in binucleation and tetraploidy^47^. The mice undergo significant dietary changes at this time as they transition from milk to chow^44^,^48^. Considering this, it is tempting to speculate that nutritional and hormonal variations between birth and weaning may serve as natural signals to initiate the postnatal splicing shift by modulating expression/activity of specific-splicing factors^9^,^49^.

**Figure 8:**
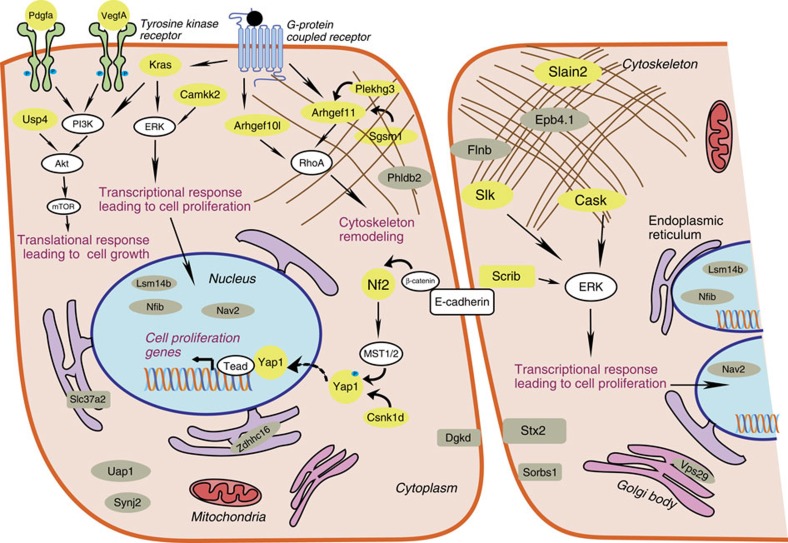
ESRP2 controls AS of cell proliferation and differentiation-related genes. Schematic diagram illustrating the network of proteins containing ESRP2-regulated alternative exons. ESRP2 targets in yellow bubbles are known to play roles in cell growth and proliferation by interacting with other cellular proteins (white bubbles). ESRP2 targets in grey bubbles play roles in other cellular functions including cell differentiation and adhesion.

Our results establish ESRP2 as a key hepatocyte factor, which controls up to 20% of splice isoform transitions occurring naturally during the postnatal period of liver development. ESRPs are epithelial-specific regulatory factors that bind to GU-rich motifs on pre-mRNAs and modulate splicing of alternative exons in a position-dependent manner^41^,^42^,^43^. While previous studies have focused on ESRP1, we demonstrate that ESRP2 is the sole paralogue expressed in human and mouse livers and that it controls a highly conserved splicing regulatory network to facilitate terminal differentiation and postnatal maturation of hepatocytes. The gain- and loss-of-function studies reveal that not only is ESRP2 necessary and sufficient for these AS transitions, but that there are detrimental functional consequences to the liver in its absence. For instance, characterization of *Esrp2* KO mice revealed defects in cell proliferation, hepatic zonation and albumin production. Consistent with these phenotypes, we find that many of the ESRP2 target exons are encoded in genes that control cell growth and proliferation, cell–cell adhesion and cell differentiation (Fig. 8). Amongst these are major components of Hippo (*Nf2, Csnk1d, Yap1*) and Rho/Ras GTPase (*Arhgef10l*, *Arhgef11*, *Plekhg3*, *Kras, Sgsm1*) signalling pathways as well as gene products involved in PI3K/AKT (*Pdgfa*, *Vegfa*, *Usp4, Kras*) and MAPK/ERK (*Slk*, *Camkk2*, *Scrib*, *Cask*) signalling pathways^50^,^51^,^52^,^53^ (Fig. 8). Future studies will examine the functional roles of individual ESRP2 target transcripts and how their splice isoform switching affects hepatocyte proliferation, differentiation, and maturation.

## Methods

### Animal models and human samples

Livers, non-parenchymal cells and hepatocytes at specified time points were isolated from FVB/NJ wild-type mice. We followed the National Institutes of Health (NIH) guidelines for use and care of laboratory animals and all experimental protocols were approved by IACUC (Institutional Animal Care and Use Committee at University of Illinois, Urbana-Champaign and University of Pennsylvania). Whole liver tissues, hepatocytes and non-parenchymal cells were isolated from FVB/NJ mice postnatal day 0 (P0) and adult stage (3 months. male) for protein and RNA isolation. Whole liver tissues were isolated from 4-month old adult *Esrp2* KO and wild-type mice (males and females) for protein and RNA isolation as well as histological studies. All animals used in this study, except P0 pups, were ear tagged and given specific identification numbers. Only ear tag numbers were used to identify the individual animals at the time of performing histological studies, serum biochemistry and liver function tests to keep the study double blinded. The animals were grouped as wild-type or *Esrp2* KO, without randomization, as per their genotyping information when analysing the data from the above mentioned tests. This study is not gender specific and includes both male and female mice. Human fetal (22-week old) and adult (51-year-old Caucasian male) liver RNAs were purchased from Clonetech Laboratories, Inc.

### RNA-seq analysis

Total liver RNAs from different developmental stages of wild-type (FVB/NJ) mice were isolated using RNeasy tissue mini-kit (QIAGEN). RNA quality was measured using an Agilent Bioanalyzer and RNA was quantified using Qubit Fluorometer (Life Technologies) before library generation. Hi-Seq libraries were prepared, and paired-end 100 bp Illumina sequencing was performed by the Genomic and RNA Profiling Core (GARP) at Baylor College of Medicine as previously described^33^. RNA-Seq reads were aligned to mouse genome and transcriptome using a previously described method^54^,^55^. Mapping percentage and details of each of the samples are shown in Supplementary Table 1. Gene expression levels were determined using RPKM (Reads Per Kilobase of transcript per Million mapped reads). Genes with significant expression difference between two groups of samples, false discovery rate (FDR)<0.1) was determined using DESeq^56^. Differential splicing events between two groups of samples were identified using MATS (FDR≤0.1, ΔPSI≥15%)^57^. To identify significantly different 3′ UTR usage between two groups (FDR<0.1), core and extension regions of tandem 3′ UTRs were identified based on RefSeq annotations and *P* values were calculated using the numbers of reads mapped to the core and extension regions of two groups using Fisher’s exact test. A minimum read coverage of 5 was required.

### Motif analysis

Motif analysis was carried out for four intronic regions flanking the exon of interest. Up to 250 bases away from the corresponding exon–intron boundary were included. For introns shorter than 500 bases, only half of the intronic regions were used. Similar methods as previously described^26^ were used for motif analysis and briefly described.

For motif conservation, we analysed sequence conservation of pentamers in the mouse intronic regions to identify potential splicing regulatory elements. The mouse introns were aligned to seven other mammalian genomes that have at least five sequence coverage in the UCSC 28-way multigenome alignment^58^. For each pentamer in each region, a conservation rate was calculated as the fraction of aligned and conserved occurrences among total occurrences. The significance of conservation rate of each pentamer is evaluated by comparing ten other pentamers with similar expected conservation rate calculated using the first-order Markov model. This procedure essentially controls for possible sequence bias in the dataset. *P* value was calculated by using the binomial distribution. For motif enrichment, in order to account for background sequence biases, the introns corresponding to each region were binned according to their GC frequency into ten groups. Expected pentamer frequency was calculated for each pentamer by using the first-order Markov model in introns of each GC group. Pentamer enrichment was then evaluated by comparing the occurrence frequency of each pentamer to the overall expected frequency calculated by summing up the expected counts of all GC groups. *P* value was calculated by using the binomial distribution.

### Gene ontology and pathway analysis

Gene ontology analysis was performed using DAVID as previously described^59^. Mouse reference genome was used and three gene ontology terms were used (BP, CC and MF) and three pathways were used (Biocarta, Kegg and Panther) for the analysis. Functional annotation clustering was performed and the top clusters (enrichment score=*P* value <0.05) were summarized. For the ESRP2 targets, the top individual gene ontology terms (*P* value <0.01) were used. The ClueGo plug-in^60^ from Cytoscape^61^ was utilized to create pathway and gene ontology enrichment network for ESRP2 targets. Gene ontology databases for Biological Processes, Molecular Functions and KEGG were included in the analysis (enrichment score=*P* value<0.01). A list of sequence specific, mouse DNA- and RNA-binding proteins was downloaded from the AmiGO2 database. Genes were then cross-referenced with the RNA-seq data to determine those changing >3-fold in expression during mouse liver development.

### Generation of *Esrp2* KO mice

The *Esrp2* KO allele was generated as part of the Knockout Mouse Project (KOMP) and purchased from Velocigene (C57BL/6N-Esrp2tml(KOMP)^vlcg^). The *Esrp2* gene locus was replaced by a LacZ and a floxed neomycin selection cassette. ES clone AG3 was injected in to Balb/c blastocyctes by the University of Pennsylvania Transgenic and Chimeric Mouse Facility, and resulting chimeras were crossed to C57BL/6J females for germline transmission. Genotypes were verified by tail biopsy PCR (Primers: E2 Common F: 5′-CGCGGGCGGGTCTCTGC-3′, E2 wild type R: 5′-CTCCCCTCCCCCTCGAAGTAGTGT-3′, and E2 KO R: 5′-CAAATCTCCACTCCCCGTTCAAAG-3′) (wild type: 638 bp, KO: 337 bp). Heterozygous *Esrp2*^*+/−*^ mice were crossed to generate *Esrp2*^*−/−*^ KO mice.

### Isolation of adult hepatocytes and non-parenchymal cells

Hep and NPC fractions were isolated with modifications of previously described methods^62^. Briefly, male adult FVB/NJ wild-type mice were perfused with 50 ml of Solution A (0.5 M EDTA in 1 × Hanks Balanced Salt Solution without Ca^2+^ & Mg^2+^). Following this, the livers were perfused using 50 ml of Solution B (3,000 U of collagenase type I from Worthington, 0.54 M CaCl_2_ and 40 mg ml^−1^ soybean trypsin inhibitor in 1 × Hanks Balanced Salt Solution with Ca^2+^ and Mg^2+^). The perfused liver was removed in a Petri dish containing 1 × PBS and cell scrapers were used to remove loose cells. The crude cell prep was filtered through 100 μm mesh filter and the resulting cell suspension was centrifuged at 50*g* for 5 min. The Hep pellet was further washed with 1 × PBS twice. The NPCs, which are in the supernatant, were isolated by centrifugation at 320*g* for 10 min and the resulting NPC pellet was washed with 1 × PBS twice.

### Isolation of P0 hepatocytes and non-parenchymal cells

The P0 livers were collected from FVB/NJ wild-type pups (males and females) and minced in Solution A. The tissue fragments were then agitated in Solution A at 37 °C for 20 min. The supernatant was discarded and the minced tissue was now digested using Solution B for 30 min at 37 °C. The resulting crude liver preparation was filtered using 100 mm mesh filter. The resulting cell suspension was processed as above for obtaining Hep and NPC fractions. Purified cell fractions from P0 and adult stage were subsequently lysed to extract protein or RNA using standard procedures.

### Protein isolation and western blot analysis

Proteins were isolated by homogenizing frozen liver tissue or purified cell fractions with cold homogenization buffer, 400 μl per 100 mg liver tissue (HEPES-KOH, pH 7.5, 10 mM, Sucrose 0.32 M, MG132 5 μM, EDTA 5 mM, Proteinase inhibitor (1/2 tablet per 10 ml buffer)). Samples were sonicated and clarified by centrifugation and protein content measured using BCA protein assay kit (Thermo Scientific). A total of 40–60 μg proteins were resolved on 10% SDS–polyacrylamide gel electrophoresis gels and transferred onto PVDF membranes (Immobilon, Millipore). Membranes were blocked in Tris-buffered saline (TBS) containing 10% non-fat dry milk and 0.1% Tween 20 (TBST), prior to incubation with primary antibody (0.5–2 mg ml^−1^ dilution) overnight at 4 °C. The membranes were then washed with TBST followed by incubation with an appropriate horseradish peroxidase-conjugated secondary antibody for 2 h. The immunoreactivity was visualized on ChemiDoc XRS+ using the Clarity Western ECL kit (BioRad). HRP-conjugated rabbit monoclonal anti-ESRP1/2 (78 kDa; Rockland, 23A7.C9), mouse polyclonal anti-E-cadherin (135 kDa; BD Biosciences, 610181), goat polyclonal anti-vimentin (51 kDa; Abcam, Ab11256), chicken polyclonal anti-albumin (66 kDa; Abcam, Ab106582), mouse monoclonal anti-N-cadherin (140 kDa; Life Technologies, 33–3900), goat polyclonal anti-pRB-Ser 780 (110 kDa; Santa Cruz, SC12901), rabbit polyclonal anti-Cyclin D1 (36 kDa; Cell Signaling, 2922S), mouse monoclonal anti-TBP (38 kDa; Pierce, 51841), MBNL2 (40 kDa; gift from T. Cooper), goat polyclonal anti-Gapdh (36 kDa; Santa Cruz, 20357) and goat polyclonal anti-β-actin (40 kDa; Santa Cruz, SC1616) were used. HRP-conjugated goat anti-mouse IgG light chain-specific (BioRad, 1706516), goat anti-rabbit IgG (Thermo, 31460), rabbit anti-goat IgG (Santa Cruz, sc-2922) and rabbit anti-chicken IgY (Pierce, 31401) secondary antibodies were used at 1:2000 to 1:5000 dilutions. Uncropped images of immunoblots are shown in Supplementary Fig. 7.

### Estimation of serum albumin

Total proteins in serum of wild type (*n*=6) and *Esrp2* KOs (*n*=11) were determined by BCA protein assay kit (Thermo Scientific). A total of 10 μl of diluted (1:50 in 1 × PBS) serum samples were used to perform a Western blot for albumin. The intensities of the bands obtained were quantified by ImageLab software (BioRad). Serum albumin per mg of protein was calculated by using the following formula: (intensity of albumin band × 1,000)/(total amount of serum loaded).

### Estimation of albumin in *Esrp2* KO and wild-type livers

Total proteins were isolated from livers of wild-type (*n*=2) and *Esrp2* KOs (*n*=4) mice and concentration was determined by BCA protein assay kit (Thermo Scientific). A total of 50 μg of the above proteins were used to perform a western blot for albumin. The intensities of the bands obtained were quantified by ImageLab software (BioRad). Albumin per mg of protein was calculated by using the following formula (intensity of albumin band × 1,000)/(total amount of protein loaded (that is, 50 μg)). The results for these were represented as box and whisker plots.

### Gene expression and splice isoform analysis

Total RNAs were isolated from mouse livers or purified cell fractions using TRIzol reagent. Upon DNAse treatment (Promega), RNAs (∼5 μg) were reverse transcribed using random hexamer primers and Maxima Reverse Transcriptase kit (Thermo Scientific). The cDNA was diluted to 25 ng μl^−1^ with nuclease free water and used for alternative splicing or qRT–PCR assays as previously described^26^. PSI values for the variably spliced region were calculated with ImageLab software (BioRad) as ((exon inclusion band intensity)/(exon inclusion band intensity+exon exclusion band intensity) × 100). qRT–PCR was performed in triplicate using 50 ng of cDNA per reaction on an Eco Real-Time PCR system (Illumina) using PerfeCTa SYBR Green FastMix (Quanta). *Esrp1* and *Esrp2* qRT–PCR assays were performed with predesigned TaqMan primers and probes according to the manufacturer’s instructions (Applied Biosystems). An initial activation step for 10 min at 95 °C was followed by 40 cycles of 95 °C for 10 s and 60 °C for 30 s. Details of the primer sequences are can be found in Supplementary Table 2. Fold change of the mRNA was calculated as previously described^26^.

### *In situ* hybridization

Plasmids for *in situ* hybridization were produced by inserting Hind II and EcoRI digested amplified PCR products for *Esrp2* cDNA in pDP19 vector (Ambion). Sense and anti-sense RNA strands were generated as previously described^43^. *In situ* hybridization for *Esrp2* was performed by Phylogeny Inc. (Columbus, Ohio.) The C57BL/6 mouse samples were prepared for *in situ* hybridization as previously described.

### Histology and immunohistochemistry

Liver tissues from wild-type (*n*=3) and *Esrp2* KO mice (*n*=3) were harvested and fixed overnight in 10% neutral-buffered formalin, and then embedded in paraffin and sectioned (5 μm thickness). Hematoxylin and eosin (H&E) and trichrome staining were performed using standard histological methods. For immunohistochemistry, unstained slides were deparaffinized in xylenes (two treatments, 5 min each), rehydrated sequentially in ethanol (2 min in 100%, 2 min in 95% and 2 min in 80%), and washed for 3 min in water. Antigen retrieval was performed by boiling the sections in sodium citrate buffer (10 mM sodium citrate, 0.05% Tween 20, pH 6.0) for 20 min at 100 °C and cooled for 20 min. Endogenous peroxidase activity was quenched with a solution of 3% hydrogen peroxide solution (Fisher Scientific). After washing, sections were blocked (2% normal goat serum, 1% bovine serum albumin (BSA), 0.1% Triton X-100, 0.05%Tween 20 in 1 × PBS) for 30 min and incubated with primary antibodies against E-cadherin (1:500, BD Biosciences 610181), Ki-67 (1:500, BD Biosciences 550609), phospho-HIST3H3 Ser10 (1:100, Millipore 06–570), glutamine synthetase (1:1000, BD Biosciences 610518), PCK1 H-300 (1:50, Santa Cruz SC32879) or CYP2E1 (1:100, Millipore AB1252) at 4 °C for 12 h. After several washes, sections incubated with HRP-conjugated goat anti-mouse IgG light chain-specific antibody for 2 h. For visualization of signal, DAB kit (Vector Labs) was used according to the manufacturer’s instructions. All intermediate washing steps were done using 0.1 M PBS, 0.5% Tween 20, pH 7.2, and all antibodies were diluted in 1 × PBST with 1% bovine serum albumin. Slides were sealed with a coverslip after lightly counterstaining with hematoxylin and photographed with an EVOS XL microscope.

### Quantification of cell area, ploidy, apoptotic and proliferating cells

Cell area of wild-type and *Esrp2* KO liver sections was analysed by wheat germ agglutinin (WGA) staining. Sections were dewaxed and washed with 1 × PBS and incubated with 10 μg WGA-Alexa Fluor 488 for one hour at room temperature followed by additional washes with 1X PBS. Slides were mounted with DAPI containing hardset Vectashield mounting media (Vector Labs) and sealed with coverslips. The average cell area was quantified by choosing five random fields from each section and determining cell area for each cell in the field using ImageJ software (http://imagej.nih.gov/ij/) for wild-type (*n*=3) and *Esrp2* KO sections (*n*=3). TUNEL assay was performed using fluorescein *in situ* cell death detection kit (Roche) to evaluate apoptosis. The same software and method of quantitation was used to enumerate total number of cells, mono and binucleated cells, apoptotic cells, pHIST3H3 and Ki-67 positive cells per field quantitation.

### Liver function tests

Blood from wild-type (*n*=6) and *Esrp2* KO (*n*=11) mice was collected by retro-orbital puncture in Capiject gel/clot activator tubes and centrifuged for 10 min at 7500*g*, 4°C, and stored at −80 °C until further analysis. Serum chemistry analyses of alanine aminotransferase, aspartate aminotransferase, cholesterol and triglycerides were performed using specific assay kits and following the manufacturer’s protocols (Thermo Scientific). Random and steady-state glucose measurements were done by collecting blood from wild-type (*n*=8) and *Esrp2* KO (*n*=8) mice and detection using OneTouch Ultra 2 Blood Glucose Monitoring System. For fasting glucose levels, the mice were fasted for eight hours before measurement of glucose levels.

### Adenovirus production and cell culture

HepG2 and AML12 cell lines were obtained from ATCC (Catalogue no. HB-8065 and CRL-2254, respectively) and cultured according to specifications as stated by ATCC. When tested for mycoplasma contamination (Biotool, catalogue no. B39032) prior to use, these cell lines tested negative. cDNAs encoding FLAG-tagged mouse *Esrp2* (ref. 43) were sub-cloned into the p-Adeno-X-ZsGreen1 vector (Clonetech, 632267) using the In-Fusion kit (Clonetech, 639646) as per the manufacturer’s instructions. High-titre adenoviruses were generated by transfecting Ad-293 cells (∼70% confluent) in T-25 flasks with linearized recombinant adenoviral plasmid using Mirus TransIT-2020 kit and cells were harvested once cytopathic effect was seen. Following this, two viral amplification steps were performed and the viral particles were purified using CsCl gradient as mentioned in the Adeno-X Adenoviral System 3 user manual. After purification of viral particles, the titre was determined by ultraviolet spectrophotometry at 260 nm. To determine the effect of overexpression of ESRP2 in HepG2 and AML12 cells, T-25 flasks containing ∼50% confluent HepG2 and AML12 cells were infected with 1.5 × 10^9^ o.p.u. (optical particle units) of the ESRP2 or GFP adenovirus for 48 h and cells were harvested to extract RNA and protein for further analysis.

### Statistical analyses

Results are expressed as mean±s.d., unless otherwise specified. Statistics were performed using the GraphPad Prism 6 software. Statistical significance was determined using two-tailed Student’s *t*-test (*P*<0.05). Correlation between *Esrp2* KO and overexpression samples was carried out using Pearson’s product–moment correlation. The conservation of ESRP2 AS targets between mouse and humans was carried out using two-sample test for equality of proportions with continuity correction.

## Additional information

**How to cite this article:** Bhate, A. *et al*. ESRP2 controls an adult splicing programme in hepatocytes to support postnatal liver maturation. *Nat. Commun*. 6:8768 doi: 10.1038/ncomms9768 (2015).

## Supplementary Material

Supplementary Information Supplementary Figures 1-7, Supplementary Table 1-2 and Supplementary Reference.

Supplementary Data 1 RT-PCR validation of alternative splicing in mouse liver development.

Supplementary Data 2 Conservation of alternative splicing between humans and mice.

Supplementary Data 3 Cell-type-specific alternative splicing in mouse liver development.

Supplementary Data 4 Splicing quantification of ESRP2 regulated events.
