# Supplementary material for: ESRP2 controls an adult splicing programme in hepatocytes to support postnatal liver maturation
Source: Nat Commun. 2015 Nov 4;6:8768. doi: 10.1038/ncomms9768 (PMC4635967; doi:10.1038/ncomms9768)
Supplement: Supplementary Information — Supplementary Figures 1-7, Supplementary Table 1-2 and Supplementary Reference. [file ncomms9768-s1.pdf]

Supplementary Information

Supplementary Figure 1.

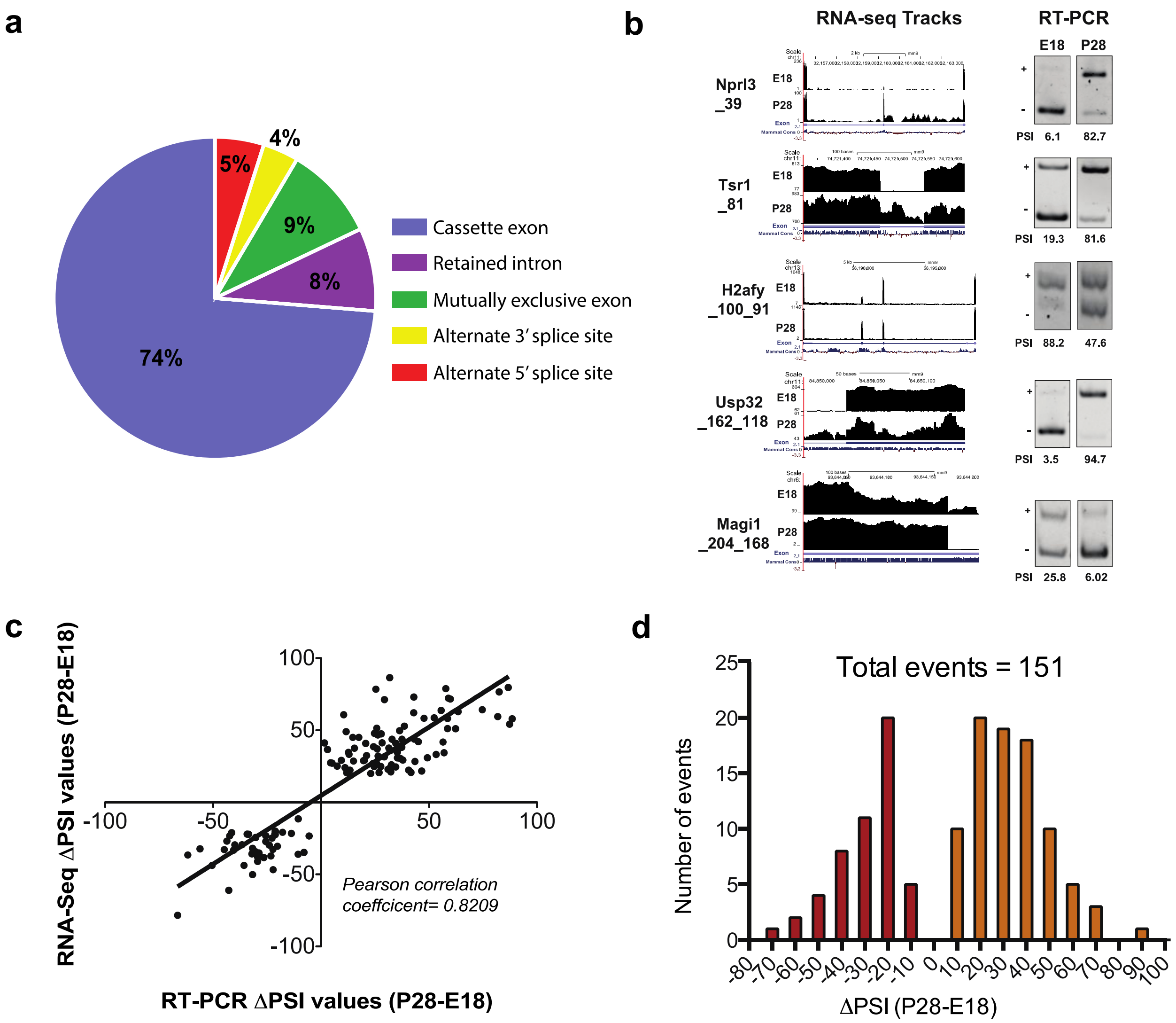

Supplementary Figure 1. Validation of alternative mRNA splicing during mouse liver development

(a) Pie chart of the different types of alternative splicing (AS) events analyzed in this study. **(b)** Left panel shows RNA-seq data displayed on the UCSC genome browser for each event. Right panel shows representative gel images of RT-PCR validation of the same AS events. The bands corresponding to (+) indicate exon inclusion and (-) indicates exon exclusion. E18 corresponds to embryonic day 18 and P28 corresponds to postnatal day 28 liver samples. **(c)** Scatter plot showing comparison of RT-PCR and RNA-seq based ΔPSI (Difference in Percent Spliced In) values for 179 events. **(d)** Frequency Distribution table of exon inclusion and skipping events based on the ΔPSI (P28-E18) values.

Supplementary Figure 2.

a

Regulation Pattern	% Sequence Similarity	Variable Region in frame
Mouse (>20%), Human (>10%)	84	40/55
Mouse (>20%), Human (<10%)	90	28/33
Mouse (>20%), Human single isoform	78	26/39

b

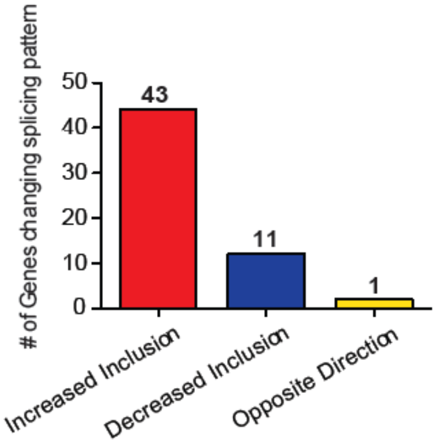

Supplementary Figure 2. Conservation of splicing in mouse and humans during liver development
(a) The table represents average sequence similarity for each of the three splicing categories and the number of exons that maintain the original frame of translation in mouse and human AS during liver development. **(b)** Direction of splicing for the 55 events that showed regulation in both mouse and human.

Supplementary Figure 3.

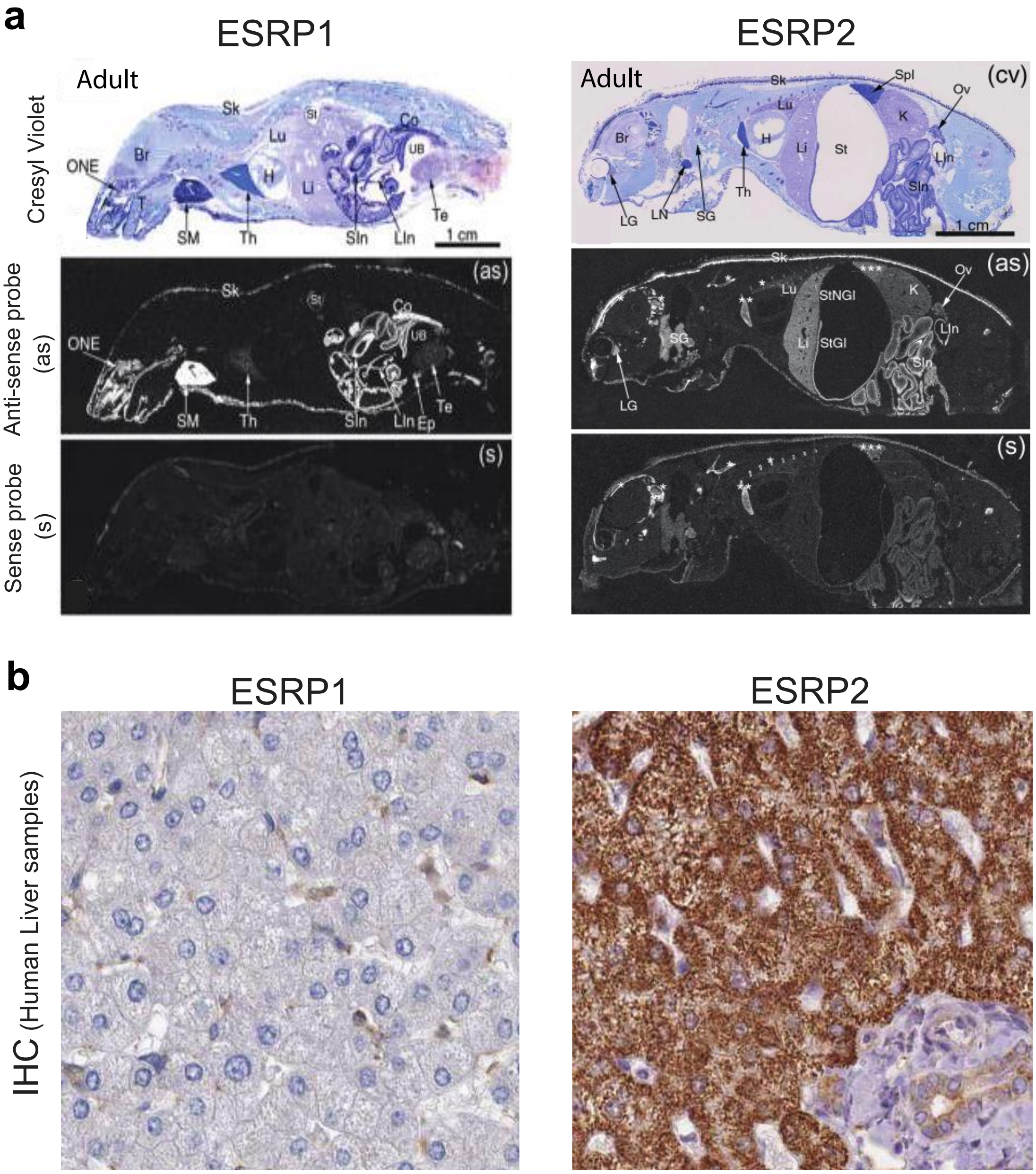

Supplementary Figure 3. Expression of ESRP1 and ESRP2 in various tissues
(a) In-situ hybridization analysis demonstrating the expression of ESRP1 (left panel; obtained from Warcheza et al. 2009) and ESRP2 (right panel) in various mouse tissues by X-ray film autoradiography detection of ESRP1 and ESRP2 mRNAs. Abbreviations: Br –brain; Co – colon; Ep – epididymis; H – heart; K – Kidney; LG- Lacrimal Gland; Li – liver; LIn – large intestine; LN- Lymph node; Lu – lung; ONE –olfactory neuroepithelium; Ov - ovaries; SG- salivary Gland; Sk – skin; SIn – small intestine; SM – submaxillary gland; Spl – spleen; St – stomach; T – tongue; Te – testis; Th –thymus; UB – urinary bladder. **(b)** IHC staining of ESRP1 and ESRP2 in human liver tissue images obtained from The Human Protein Atlas (www.proteinatlas.org[1]).

Supplementary Figure 4.

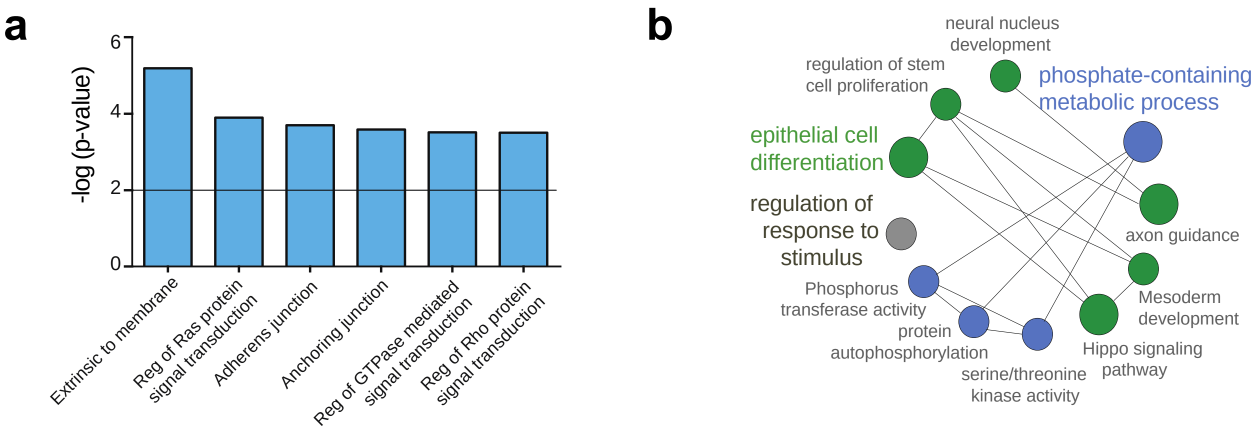

Supplementary Figure 4. Analysis of the ESRP2 regulated splicing network in the liver
(a) Gene Ontology analysis of ESRP2 target genes. **(b)** Protein-protein interaction analysis of ESRP2 target genes.

Supplementary Figure 5.

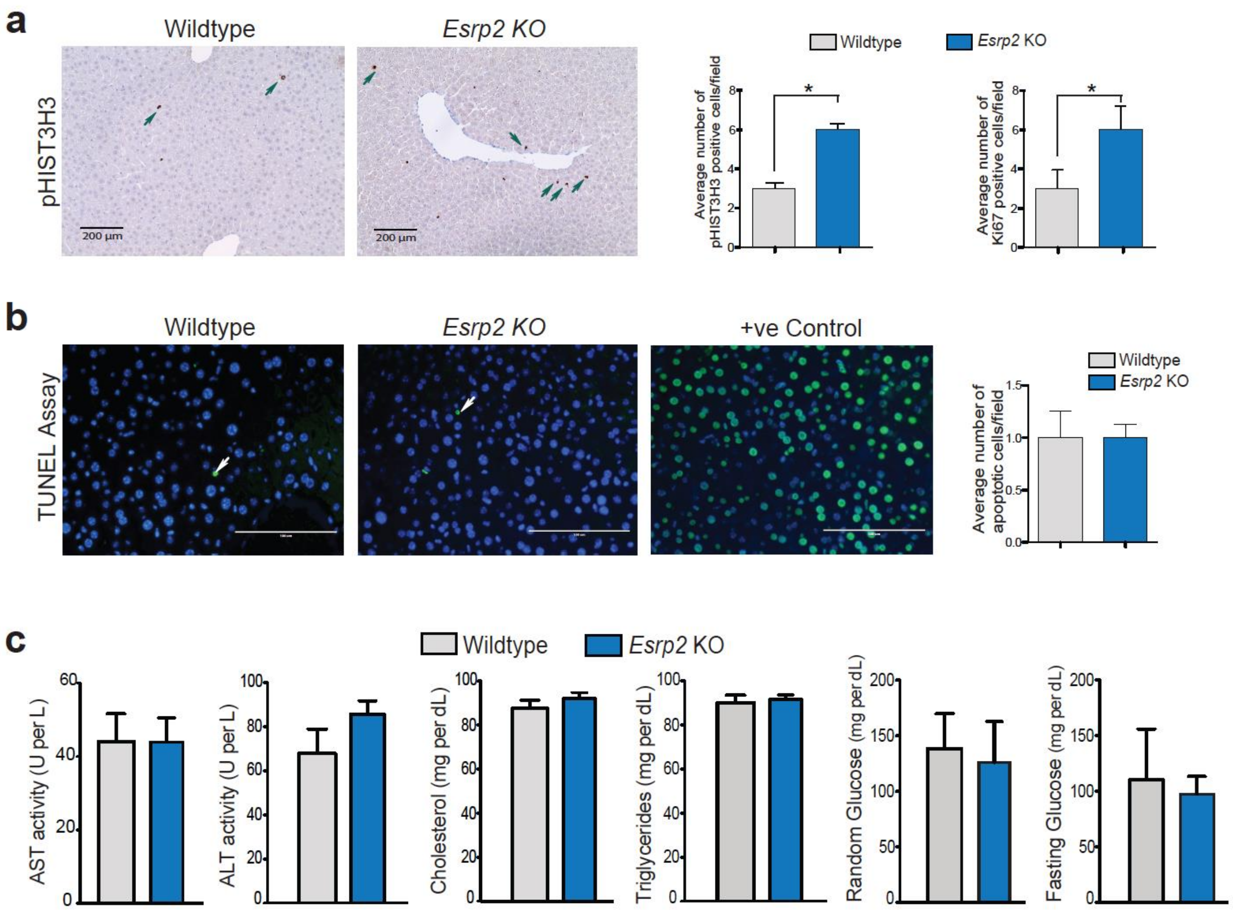

Supplementary Figure 5. Phenotypic characterization of *Esrp2* KO mouse
(a) Increased number of phospho-histone 3 (pHIST3H3) positive cells in *Esrp2* KO livers (green arrows), Scale bars, 200 µm; quantification of Ki-67 and pHIST3H3 positive cells. mean ± SD **(b)** No significant difference in apoptosis between WT and *Esrp2* KO animals as shown by TUNEL assay, Scale bars, 100 µm. White arrows point to apoptotic nuclei. Bar graph indicates the quantification of average number of apoptotic cells per field in WT and *Esrp2* KO sections. mean ± SD **(c)** Blood serum levels of ALT, AST, cholesterol, triglycerides, random and fasting glucose levels of WT and *Esrp2* KO mice. mean ± SD , P<0.005, Student's t-test

Supplementary Figure 6.

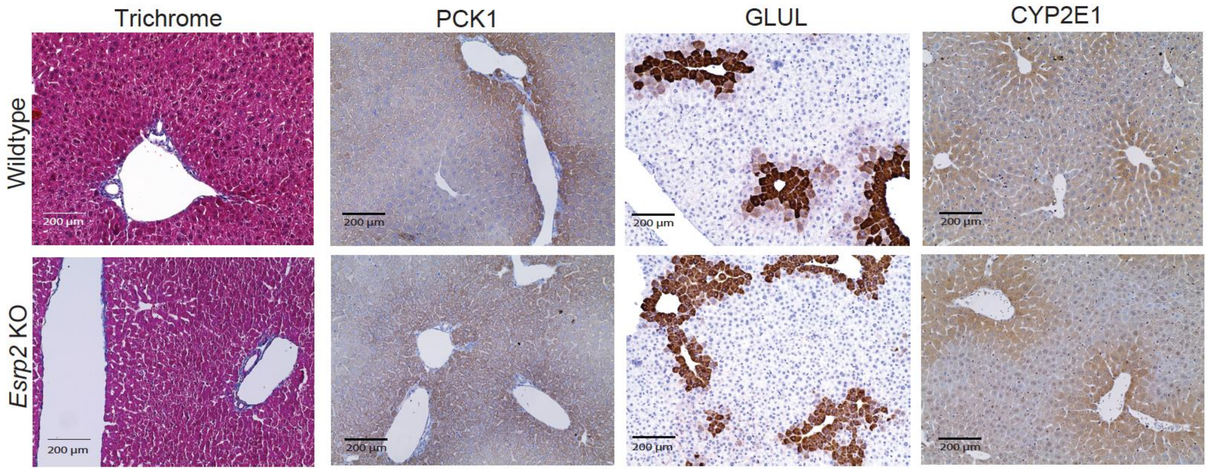

Supplementary Figure 6. Characterization of liver zonation in *Esrp2* KO mice
No apparent fibrosis in WT or *Esrp2* KO animals as evidenced by trichrome staining; reduced and diffused periportal marker phosphoenolpyruvate carboxykinase (PCK1) staining in *Esrp2* KO compared to WT; no difference in perivenous staining of glutamine synthetase (GLUL); slightly diffused cytochrome P450 2E1 (CYP2E1) staining in perivenous region in *Esrp2* KO compared to WT. Scale bars, 200 µm

Supplementary Figure 7.

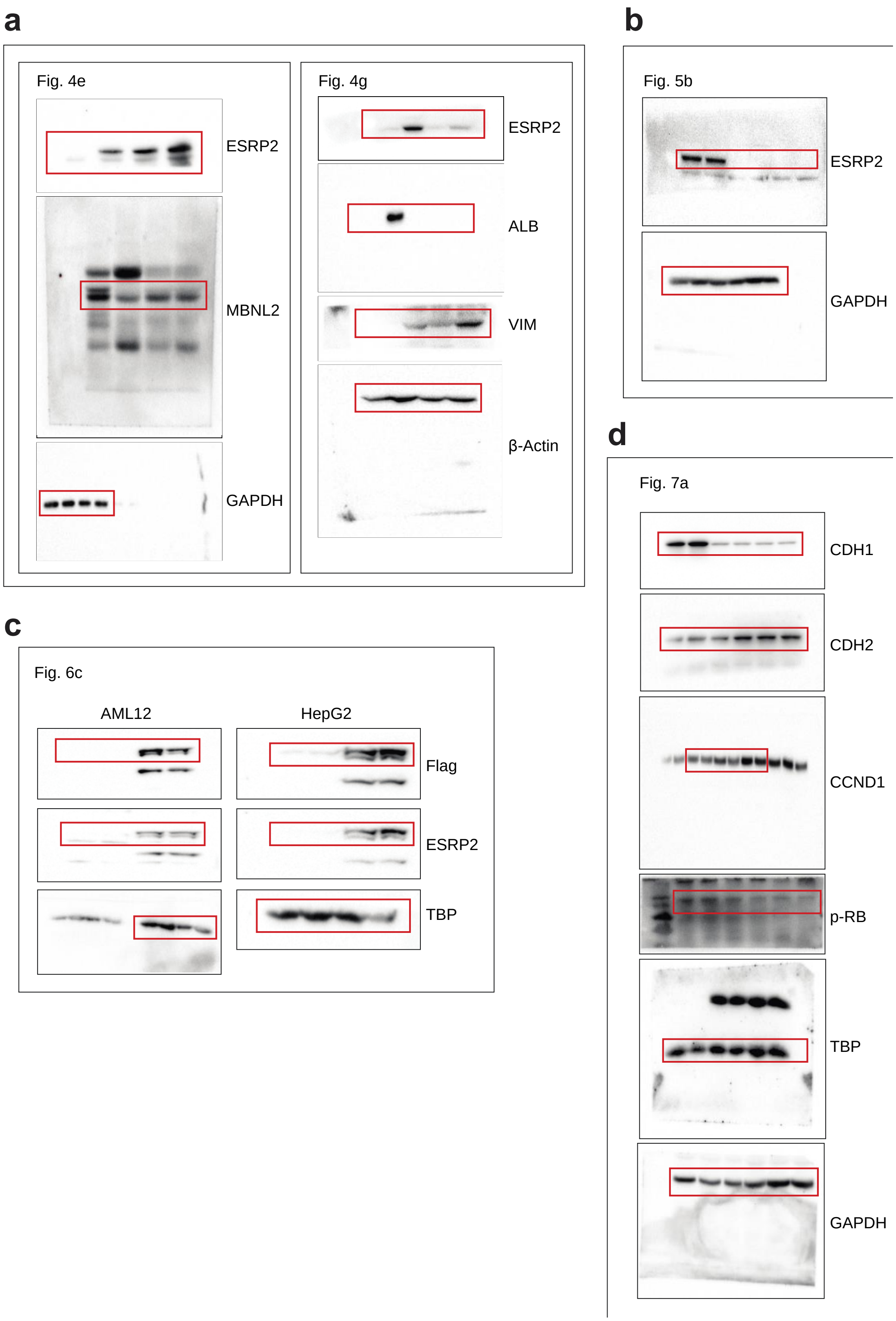

Supplementary Figure 7. Uncropped images of immunoblots shown in Fig. 4 to Fig. 7

Uncropped images of immunoblots displayed in **(a)** Fig. 4e and f; **(b)** Fig. 5b; **(c)** Fig. 6c; **(d)** Fig. 7a are presented here. Red boxes indicate the lanes used in the Figures.

Supplementary Table 1: RNA-Seq Analysis

Sample Name	Read length	Total reads	Uniquely mapped reads	Mapping rate
**Liver embryonic day 18 rep1**	101x2	3,718,224	3,255,332	87.5%
**Liver embryonic day 18 rep2**	101x2	3,555,666	3,136,002	88.2%
**Liver postnatal day 14 rep1**	101x2	3,220,400	2,856,919	88.7%
**Liver postnatal day 14 rep2**	101x2	3,795,466	3,313,816	87.3%
**Liver postnatal day 28 rep1**	101x2	3,618,022	3,131,980	86.5%
**Liver postnatal day 28 rep2**	101x2	3,569,840	3,147,281	88.1%
**Liver Adult rep1**	101x2	3,616,912	3,207, 804	88.6%
**Liver Adult rep2**	101x2	3,640,783	3,233,538	88.8%

Supplementary Table 2: Primer Sequences

Gene name	Primer Sequence (5'-3')		Amplicon Size
	Forward	Reverse	
**Mouse Primers for qRT-PCR assays**			
Celf1	CAGATTGAAGAGTGCCGGATA	TAGCTGTCTGTGCCATGGTT	97
Celf2	ACTTGGGGGAACCTAACAGG	CTGAATGCCACTGAATGCAC	105
Esrp2	TATAAAGCCACAGGGGAGGA	TCTTCCCGTGATAGGAAACG	79
Hnrnp A	ATTTTGGTCGAGGAGGGAAC	ATTATAGCCATCCCCACTGC	92
Hnrnp H	GCAGAGGAGCTGGTTTTGAG	GAACCAAATCCATAGCCATCA	102
Hnrnp L	CTGCTTGTATGGCAATGTGG	AGCATAGCCATCAGCCATTT	85
Hnrnp LL	CTGGCTCCGTTGTAATGGTT	GTGCCAGGAATGGTCTTCAT	124
Mbnl1	GGAGTTCCAGTGCCAGCAG	CACGCTGGTACTCTCGACAC	88
Mbnl2	GGCTCAACTGCAACTCAGAA	AGCGGCAGTCTGTCTCTCC	100
Ptbp1	CGTTCACCAAGAACAACCAG	TTGTAGATGTTCTGGCCATCC	100
Srsf1	CGCTTAGACCTTCCTACTGGT G	CCCTGCATATGGAGAGGACA	99
Srsf2	CCACCCCGTCGGTACG	CGACCTGGACCGACTCC	99
Cyp2b10	TGCTGTCGTTGAGCCAACC	CCACTAAACATTGGGCTTCCT	161
Albumin	TGCTGAGACTTGCCAAGACA	TCCATATTCTCCAAGCTTCTCGT	170
Cyp3a11	TCTCATAAAGCCCTTTCTGA	AATGCAGGGTGAAGGAAAGT	103
Fbp1	TGAGCCTTCTGCGAAGGATG	GAAGCAGTTGACACCACAAT	118
Igfr2	AGAGAGGAAGGAGACAACTG	CAAGTAGTAGGTGTACTCGC	117
Vimentin	ACCAGGTCTGTGTCCTCGTC	AATAGAGGCTGCGGCTAGTG	154
Meg3	TCCTCACCTCCAATTTCCCC	GAGCGAGAGCCGTTCGATG	71
Cyclin E	GATCGTTACATGGCATCACA	AAACTGGTGCAACTTTGGAG	120
Cyclin B2	GCCAAGAGCCATGTGACTATC	CAGAGCTGGTACTTTGGTGTTC	114
E2f8	GCCTCTTCCTGCCTCCTTAG	GGAGCGGAACTGATCTTCCT	159
p27	CAGAATCATAAGCCCCTGGA	GGTCCTCAGAGTTTGCCTGA	190
Cdk1	GCCAGATAGTGGCCATGAAG	TCCATGGACAGGAACTCAAA	178
Cdk6	AATCTGCTCAACCCATCGAG	GTTGGATGGCAGGTGAGAGT	186
Aqp7	AAGTGTTCAGAGCCGGAAAC	GGGTGAATTAAACCCAGGTA	100
Gapdh	AACGACCCCTTCATTGAC	TCCACGACATACTCAGCAC	191
Beta-Actin	CCCTAAGGCCAACCGTGAAA	CGGAGTCCATCACAATGCCT	134
**Human Primers for qRT-PCR assays**			
**Gene name**	**Primer Sequence (5'-3')**		**Amplicon**
	**Forward**	**Reverse**	**Size**
CELF1	CAGACGGCTATCAAGGCAAT	TGGGCCATTCTCTTCTGTTC	113
CELF2	CACCAATGCAAACCCTCTCT	CGAGAGAGGTCAAGGAGTTCA	119
ESRP2	CCCTACATGCTCTGCACTGA	GGAATTCTCTTCGGAGGTCA	124
HnRNP A	AGGCAGTGGCAAGAAAAGG	CAGTTGTGGCCATTCACAGT	105

HnRNP H	AGCTGGCTTTGAGAGGATGA	TCTGACCCAAATCCATAGCC	99
HnRNP L	CTGGGGACTCGGATGACTC	ACAGGGCCACAAGGATTACA	118
HnRNP LL	GGAGGGGGAGATCGACTACT	ACGACGGGTGAAACAGAAAC	150
MBNL1	CATTTGCAAGCCAAGATCAA	AGCAGGCCTCTTTGGTAATG	129
MBNL2	GCCCAGCAGATGCAATTTAT	GGAGCAAAGCTAATAGCCGTA	108
PTBP1	GTTCGGCACAGTGTTGAAGA	CAGCAGGCGTTGTAGATGTT	135
SRSF1	GCGACATCGACCTCAAGAAT	GCAGACGGTACCCATCGTAA	126
SRSF2	GTCGACCTCCAAGTCCAGAT	TTGGATTCCCTCTTGGACAC	129
Beta-Actin	CTGGAACGGTGAAGGTGACA	AAGGGACTTCCTGTAACAATGCA	145
GAPDH	CGAGATCCCTCCAAAATCAA	GGCAGAGATGATGACCCTTT	132
**TaqMan Probes from Life Technologies**			
**Gene name**	**Catalogue Number**	**Assay ID**	**Amplicon Size**
ESRP1	4351372	Mm01220936_g1	97
ESRP2	4331182	Mm00616290_m1	68
**Primers for adenovirus cloning**			
**Gene name**	**Forward**	**Reverse**	
ESRP2	gtaactataacggtcATGGATTACAAGGATGACGATGACAAGACTCCGCCGCCGCCG	attacctctttctccCTACAAACACACCCACTCCTTAGGGGCTTG	

Supplementary reference:

1. Uhlen, M. et al. Proteomics. Tissue-based map of the human proteome. *Science* **347**, 1260419 (2015).
